# Humoral and cell-mediated immune responses to H5N1 plant-made virus-like particle vaccine are differentially impacted by alum and GLA-SE adjuvants in a Phase 2 clinical trial

**DOI:** 10.1038/s41541-017-0043-3

**Published:** 2018-01-23

**Authors:** Stéphane Pillet, Éric Aubin, Sonia Trépanier, Jean-François Poulin, Bader Yassine-Diab, Jan ter Meulen, Brian J. Ward, Nathalie Landry

**Affiliations:** 1 Medicago Inc., Québec, G1V 3V9 QC Canada; 2 Research Institute of the McGill University Health Centre, Montreal, H4A 3J1 QC Canada; 3 Caprion Biosciences, Montreal, H2X 3Y7 QC Canada; 4 Immune Design, Seattle, WA 98102 USA; 5 Immune Design, San Francisco, CA 94080-7006 USA

## Abstract

The hemagglutinination inhibition (HI) response remains the gold standard used for the licensure of influenza vaccines. However, cell-mediated immunity (CMI) deserves more attention, especially when evaluating H5N1 influenza vaccines that tend to induce poor HI response. In this study, we measured the humoral response (HI) and CMI (flow cytometry) during a Phase II dose-ranging clinical trial (NCT01991561). Subjects received two intramuscular doses, 21 days apart, of plant-derived virus-like particles (VLP) presenting the A/Indonesia/05/2005 H5N1 influenza hemagglutinin protein (H5) at the surface of the VLP (H5VLP). The vaccine was co-administrated with Alhydrogel^®^ or with a glucopyranosyl lipid adjuvant-stable emulsion (GLA-SE). We demonstrated that low doses (3.75 or 7.5 μg H5VLP) of GLA-SE-adjuvanted vaccines induced HI responses that met criteria for licensure at both antigen doses tested. Alhydrogel adjuvanted vaccines induced readily detectable HI response that however failed to meet licensure criteria at any of three doses (10, 15 and 20 μg) tested. The H5VLP also induced a sustained (up to 6 months) polyfunctional and cross-reactive HA-specific CD4^+^ T cell response in all vaccinated groups. Interestingly, the frequency of central memory Th1-primed precursor cells before the boost significantly correlated with HI titers 21 days after the boost. The ability of the low dose GLA-SE-adjuvanted H5VLP to elicit both humoral response and a sustained cross-reactive CMI in healthy adults is very attractive and could result in significant dose-sparing in a pandemic situation.

## Avian influenza: Viral shell vaccine elicits a rounded antiviral response

A H5N1 bird flu vaccine consisting of the virus’ protein shell could overcome current production woes. Canadian researchers, led by the Quebec-based biotech Medicago, constructed a plant-derived, virus-like particle vaccine that presented influenza hemagglutinin surface proteins designed to prime an immune system against future infection. When injected alongside an immunity-boosting adjuvant known as GLA-SE, the vaccine elicited levels of protective antibodies that met the requirement for EU licensing. The researchers also explain that levels of immune cells recognizing the hemagglutinin should also be considered as important indicators of H5N1 vaccine performance, as cell-based immunity plays an important part in influenza protection. Current H5N1 vaccine production is limited as the virus kills the eggs in which the progenitor virus is produced, a problem Medicago’s vaccine may be able to overcome thanks to its non-virulent virus-like particles.

## Introduction

Since the first recorded direct bird-to-human transmission of highly pathogenic avian influenza H5N1 in Hong Kong in 1997, these viruses have spread to several countries causing widespread death and illness in domestic and migratory birds as well as human infections and fatalities. Since 2003, the World Health Organization (WHO)^1^ has recorded 860 confirmed H5N1 cases with 454 fatalities (i.e., 52.8 % case-fatality rate, as of October 2017). Emergence of drug-resistant strains of avian H5N1 viruses strengthened the fact that vaccination remains the most effective public health intervention strategy and must be supported by enhanced surveillance networks. However, latest outbreaks highlighted the overall needs to improve the manufacturing capacity of influenza vaccine worldwide.^2^ Additionally, manufacturing capacity of vaccines against H5N1 viruses is limited due to the lethality of those highly pathogenic viruses to the embryonated eggs, which remains the most common producing system for influenza vaccine.^3^ Virus-like particle (VLP) expressing influenza antigenic protein can overcome most of the current pitfalls associated with traditional egg-based technologies, especially the plant-made VLP.^4–8^ Immunogenicity of influenza vaccines was historically evaluated regarding the antibody response, which remains the essential criteria for licensure. However, cell-mediated immunity (CMI) has been demonstrated to contribute significantly to the protection against influenza infection while playing a pivotal role in cross-protection and long-lasting immune response.^9–13^ We have previously demonstrated that plant-made monovalent VLP vaccines presenting influenza hemagglutinin proteins H1 or H5 induced the presence of long-term cross-reactive memory CD4^+^ T cells 6 months after immunization in healthy adults.^14^ Here we reported the short and long-term antibody responses and the CMI induced by two doses of a plant-made H5 VLP vaccine (H5VLP) adjuvanted with Alum-based (Alhydrogel^®^, Brenntag, QC) or with the synthetic toll-like receptor 4 (TLR4) agonist glucopyranosyl lipid adjuvant (GLA) formulated in a stable emulsion (GLA-SE^®^, Immune Design Corp, WA) given 21 days apart to healthy adults during a Phase II clinical trial.

## Results

**Fig. 1:**
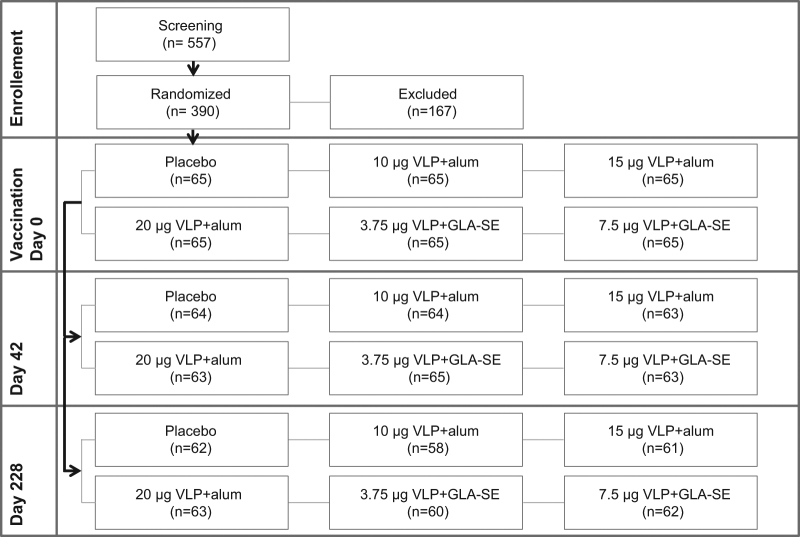
Subject disposition from screening to day 228 visit

Three hundred-ninety subjects were randomized and 97.9% of subjects completed the study through day 42 (D42) and 80% through day 228 (D228) (Fig. 1). Over 75% of the subjects were Caucasian, the remaining subjects were Asian or Black or African American (Suppl. Table 1). Gender was well distributed between groups with a slightly higher proportion of woman who received 7.5 μg of H5VLP combined with glucopyranosyl lipid adjuvant-stable emulsion (GLA-SE; 7.5 µg H5VLP + GLA group). The mean age and body mass index (BMI) were similar between groups. Twenty-five percent of subjects reported to have received an influenza vaccination in the previous year (Suppl. Table 1).

### Safety

The H5VLP influenza was generally well tolerated in all tested conditions after the 1st and the 2nd dose. Statistical comparisons for solicited symptoms showed overall significantly higher incidences in H5VLP recipient (Suppl. Figure 1). The safety profiles of alum-adjuvanted and GLA-adjuvanted groups were similar with the exception of swelling and muscle ache after the first dose. However, the majority of solicited symptoms reported in this study were mild or moderate or ≤Grade 2 intensity for all dosage regimens (data not shown).

### Antibody response

The hemagglutination inhibition (HI) titers antibody response showed that all the H5VLP + GLA groups met the European Medicines Agency’s Committee for Medical Products for Human Use (CHMP) criteria after two doses; a 87.3–93.4% of seroprotection rate (SPR), a 70.5–71.4% of seroconversion rate (SCR) and a 7.8–8.3 of geometric mean fold rise (GMFR) ranges were obtained in these conditions in which lower bound of the 2-sided 95% CI did not fall below the limit of ≥70% for SPR, ≥40% for SCR and ≥2.5 for GMFR (Table 1). The H5VLP + alum groups did not meet all the CHMP criteria, although the 15 and 20 µg doses of vaccine reached the limit for the SCR (40.0–42.4%) and GMFR (3.2–3.8) whereas SPR were just below 70% (66.7–67.8%). In addition, the 7.5 µg dose of H5VLP + GLA showed a remarkable sustained HI titers response in which all CHMP criteria were maintained 228 days after vaccination with 75.0% (62.1–85.3%), 43.3% (30.6–56.8%) and 4.2 (3.3–5.4%) fold increase of GMT level for SPR, SCR and GMFR, respectively. Overall, the H5VLP + GLA vaccine achieved all requested HI-based correlates of protection with a dose as low as 3.75 µg vaccine and allowed to maintain a high antibody response for 6 months following vaccination.

**Table 1:** Serum HI antibody response against the homologous influenza A strain at D0, D21, D42 and D228 after vaccination with adjuvanted H5VLP

Groups	*n*	Time (days post-vaccination)	GMT (95% CI)	SPR (%) (95% CI)	SCR (%) (95% CI)	GMFR (95% CI)
Placebo	64	0	9.5 (7.7–11.7)	8.1 (2.7–17.8)	–	–
	64	21	20.3 (16.0–25.8)	43.8 (31.4–56.7)	23.4 (13.8–35.7)	2.1 (1.7–2.7)
	64	42	11.9 (9.4–15.1)	20.6 (11.5–32.7)	9.5 (3.6–19.6)	1.2 (1.0–1.6)
	63	228	18.9 (15.2–23.4)	27.4 (16.9–40.2)	21.0 (11.7–33.2)	2.1 (1.6–2.7)
10 µg VLP + alum	62	0	9.4 (7.7–11.4)	8.1 (2.7–17.8)	–	–
	62	21	27.3 (20.2–36.9)	53.2 (40.1–66.0)	29.0 (18.2–42.0)	**2.9 (2.2–3.8)**
	61	42	30.3 (23.3–39.3)	57.4 (44.1–70.0)	36.1 (24.2–49.4)	**3.3 (2.5–4.4)**
	62	228	26.4 (20.6–33.8)	53.2 (40.1–66.0)	30.7 (19.6–43.7)	**2.8 (2.1–3.7)**
15 µg VLP + alum	64	0	10.4 (8.3–12.9)	12.5 (5.6–23.2)	–	–
	64	21	25.4 (20.2–31.9)	56.3 (43.3–68.6)	25.0 (15.0–37.4)	2.5 (2.0–3.1)
	64	42	33.7 (25.1–45.2)	67.8 (54.4–79.4)	**42.4 (29.6–55.9)**	**3.2 (2.4–4.3)**
	59	228	25.6 (21.0–31.3)	48.3 (35.0–61.8)	22.4 (12.5–35.3)	2.4 (1.8–3.0)
20 µg VLP + alum	64	0	10.2 (8.4–12.3)	7.8 (2.6–17.3)	–	–
	64	21	26.6 (20.3–35.0)	53.1 (40.2–65.7)	31.3 (20.2–44.1)	**2.6 (2.0–3.4)**
	64	42	39.2 (31.0–49.7)	66.7 (53.3–78.3)	**40.0 (27.6–53.5)**	**3.8 (3.1–4.7)**
	60	228	26.8 (21.3–33.5)	56.5 (43.3–69.0)	35.5 (23.7–48.7)	**2.7 (2.0–3.5)**
3.75 µg VLP + GLA-SE	63	0	9.9 (8.0–12.1)	12.7 (5.6–23.5)	–	–
	63	21	31.7 (25.4–39.6)	63.5 (50.4–75.3)	**41.3** (**29.0–54.4)**	**3.2 (2.5–4.2)**
	63	42	**81.6 (64.6–103.2)**	**87.3 (76.5–94.4)**	**71.4 (58.7–82.1)**	**8.3 (6.5–10.6)**
	63	228	32.6 (27.5–38.6)	68.3 (55.3–79.4)	33.3 (22.0–46.3)	**3.3 (2.7–4.1)**
7.5 µg VLP + GLA-SE	63	0	10.1 (8.0–12.6)	14.3 (6.7–25.4)	–	–
	63	21	33.1 (27.4–39.9)	60.3 (47.2–72.4)	33.3 (22.0–46.3)	**3.3** (2.5–4.3)
	63	42	**78.1 (64.0–95.5)**	**93.4 (84.1–98.2)**	**70.5 (57.4–81.5)**	**7.8 (6.0–10.3)**
	61	228	**43.1 (37.5–45.0)**	**75.0 (62.1–85.3)**	**43.3 (30.6–56.8)**	**4.2 (3.3–5.4)**

Geometric mean titer (GMT), percent of seroprotection rate (SPR), percent of seroconversion rate (SCR) and geometric mean fold increase ratio (GMFR) were reported. The GMT ≥ 40 and values meeting the CHMP criteria for SPR (70% of subjects with titers ≥1:32), SCR (40% of subjects with 4-fold increase of titers with ≥1:32 value) and GMFR (2.5-fold increase of GMT from Day 0) are reported in bold

### T cell response

The T cell response was assessed in ten subjects/group at day 0 (D0), day 21 (D21), D42 and D228.

#### The adjuvanted H5VLP induced a significant polyfunctional and sustained CD4^+^ T cell homologous response

**Fig. 2:**
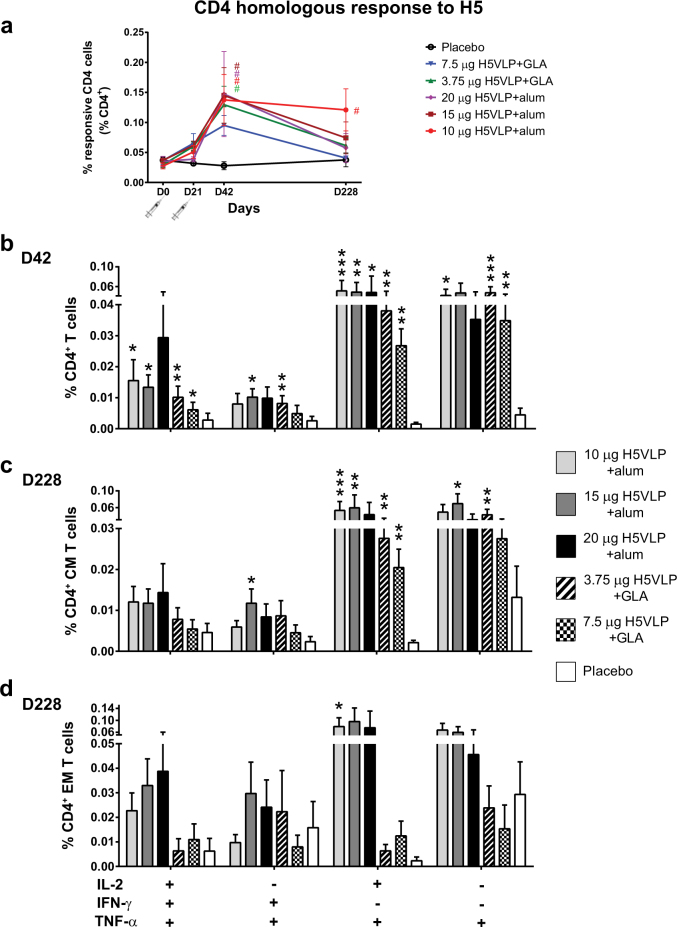
CD4^+^ homologous response to H5. The total percentages (mean ± s.e.m) of H5-specific CD4^+^ T cells secreting one or more of the cytokines IL-2, IFN-γ and TNF-α following ex vivo stimulation with H5 peptide pool before vaccination (D0) and on days 21 (D21), 42 (D42) and 228 (D228) were represented for the five vaccine regimens and Placebo (**a**). The color-matched # indicate statistically significant differences (*P* ≤ 0.05, two-way ANOVA followed by a Dunnet multiple comparison post hoc analysis, GraphPad, La Jolla, CA) with both D0 and Placebo (**a**). Effects of vaccination on the different functional signatures as defined by the expression of IL-2/IFN-γ/TNF-α in CD4^+^ T cell ex vivo stimulated with H5 peptide pool were also assessed. The percentages (mean ± s.e.m) of the significantly impacted CD4^+^ T cell functional signatures (detailed at the bottom of the figure) 42 days after prime (**b**), CD4^+^ central memory T cell signatures (CM, **c**) and CD4^+^ effector memory T cell signatures (EM, **d**) 228 days after prime were represented for the five vaccine regimens and Placebo. Asterisks indicate significant differences (*P ≤ 0.05), **P ≤ 0.01 or ***P < 0.001) from Placebo for each individual functional signature (Kruskal–Wallis test, followed by Dunn’s multiple comparisons test, GraphPad, La Jolla, CA)

The frequency of H5-specific CD4^+^ T cells secreting one or more of the cytokines interleukine-2 (IL-2), interferon-γ (IFN-γ) and tumor necrosis factor-α (TNF-α) significantly increased 21 days after the boost (D42) and was significantly higher than Placebo for all vaccine regimens with the exception of 7.5 μg H5VLP + GLA (Fig. 2a). We further detailed the impact of vaccination on all the functional signatures defined by the expression of these 3 cytokines on D42 (Fig. 2b). The frequencies of IL-2^+^/IFN-γ^+^(TNF-α^−^) CD4^+^ T cells were extremely low (<0.001%) and no differences were observed between vaccinated groups and Placebo in the rates of H5-specific single positive (SP) IFN-γ^+^(IL-2^−^/TNF-α^−^) nor IL-2^+^(IFN-γ^−^/TNF-α^−^) CD4^+^ T cells after ex-vivo stimulation at D42 (data not shown). In contrast, the frequencies of H5-specific polyfunctional triple positive IL-2^+^/IFN-γ^+^/TNF-α^+^ (TP), double positive IL-2^+^/TNF-α^+^(IFN-γ^−^) Th1 primed precursor cells (Thpp) and TNF-α^+^/IFN-γ^+^(IL-2^−^) CD4^+^ T cells significantly increased between D0 and D42 in H5VLP-vaccinated groups but not in Placebo regardless of vaccine dose or the nature of the adjuvant (Table 2). This resulted in significantly higher TP and Thpp H5-specific CD4^+^ T cells in all the H5VLP-vaccinated groups as compared to Placebo at D42, with the exception of the TP CD4^+^ T cells in the 20 μg H5VLP + alum narrowly failing to reach statistical significance (Fig. 2b). The H5-specific TNF-α^+^/IFN-γ^+^(IL-2^−^) CD4^+^ T cells were also significantly higher than Placebo in the 15 μg H5VLP + alum and 3.75 μg H5VLP + GLA groups. SP TNF-α^+^ also significantly increased between D0 and D42 in H5VLP-vaccinated groups with the exception of subjects who received 20 μg H5VLP + alum (Table 2) and the percentage of SP TNF-α^+^ CD4^+^ T cells were significantly higher than Placebo in the 10 μg H5VLP + alum and the 2 GLA-adjuvanted groups at D42 (Fig. 2b). Six months after the vaccination, H5VLP-vaccinated groups still exhibited significantly higher proportion of polyfunctional H5-specific CD4^+^/CD45RA^−^/CD27^+^ (CD4^+^ central memory, CM) and CD4^+^/CD45RA^−^/CD27^−^ (CD4^+^ effector memory, EM) T cells compared to the Placebo (Fig. 2c, d). Notably, H5-specific Thpp CD4^+^ CM T cells remained significantly higher in H5VLP-vaccinated groups than Placebo with the exception of subjects who received the highest dose of alum-adjuvanted vaccine (Fig. 2c, upper panels). The higher frequencies of TNF-α^+^/IFN-γ^+^(IL-2^−^) and SP TNF-α^+^ CD4^+^ CM T cells remained significantly greater than Placebo after 6 months in vaccinated subjects in the 15-μg H5VLP + alum and the 3.75 μg H5VLP + GLA groups, respectively. The vaccination with H5VLP also promoted H5-specific polyfunctional CD4^+^ EM T cells response particularly in alum-adjuvanted groups. Although relatively high, the frequencies of H5-specific CD4^+^ EM T cells in vaccinated groups were not significantly different from Placebo (likely due to the small number of subjects) with the exception of Thpp CD4^+^ EM T cells in the 10 μg H5VLP + alum group (Fig. 2d). Overall, alum appeared to promote higher H5-specific long-term CD4^+^ EM T cells than GLA-SE (Fig. 2d). Perforin, granzyme B and CD107a were added as markers for CD8^+^ cytotoxicity in addition to the expression of the abovementioned cytokines. However, we did not observe significant changes in the frequencies of CD8^+^ T cells expressing functional markers (data not shown).

**Table 2:** Comparison between D0 and D42 for the H5-specific or H2-specific frequencies of the principal functional signatures in vaccinated groups and Placebo

H5 peptide pool	IL-2^+^ IFN-γ^+^ TNF-α^+^ (Per 10^6^ CD4^+^ T Cells)		IL-2^−^ IFN-γ^+^ TNF-α^+^ (Per 10^6^ CD4^+^ T Cells)		IL-2^+^ IFN-γ^−^ TNF-α^+^ (Per 10^6^ CD4^+^ T Cells)		IL-2^−^ IFN-γ^−^ TNF-α^+^ (Per 10^6^ CD4^+^ T Cells)	
	D0	D42	D0	D42	D0	D42	D0	D42
10 μg H5VLP + alum	26.2	155.1**	13.9	79.6**	9.5	511.5**	66.7	419.2*
15 μg H5VLP + alum	28.2	133.6*	20.4	101.7*	24.5	487.1**	27.4	470.2**
20 μg H5VLP + alum	12.5	293.9**	5.6	98.2**	11.5	480.5**	147.0	352.5
3.75 μg H5VLP + GLA	27.7	101.3*	27.1	81.5**	15.4	380.1**	32.5	475.1**
7.5 μg H5VLP + GLA	9.1	61.1**	8.8	48.7**	21.2	268.0**	126.3	348.7**
Placebo	25.6	27.8	26.6	25.5	20.5	15.0	87.2	44.4
H2 peptide pool	IL-2^+^ IFN-γ^+^ TNF-α^+^ (per 10^6^ CD4^+^ T cells)		IL-2^−^ IFN-γ^+^ TNF-α^+^ (per 10^6^ CD4^+^ T cells)		IL-2^+^ IFN-γ^−^ TNF-α^+^ (per 10^6^ CD4^+^ T cells)		IL-2^−^ IFN-γ^−^ TNF-α^+^ (per 10^6^ CD4^+^ T cells)	
	D0	D42	D0	D42	D0	D42	D0	D42
10 μg H5VLP + alum	6.3	112.8**	12.88	68.70**	2.47	131.7**	420.7	586.9
15 μg H5VLP + alum	12.2	83.4**	17.43	105.6**	4.42	85.5**	204.8	401.6
20 μg H5VLP + alum	11.6	334.4**	12.06	113.7**	14.69	213.7*	167.0	325.6
3.75 μg H5VLP + GLA	3.8	64.7**	8.7	50.1**	7.14	120.6**	144.0	442.7*
7.5 μg H5VLP + GLA	4.4	19.3*	20.0	30.0	8.3	65.0	297.6	398.9
Placebo	11.3	9.4	12.8	9.2	5.0	9.9	103.4	181.8
H1 peptide pool	IL-2^+^ IFN-γ^+^ TNF-α^+^ (per 10^6^ CD4^+^ T cells)		IL-2^−^ IFN-γ^+^ TNF-α^+^ (per 10^6^ CD4^+^ T cells)		IL-2^+^ IFN-γ^−^ TNF-α^+^ (per 10^6^ CD4^+^ T cells)		IL-2^−^ IFN-γ^−^ TNF-α^+^ (per 10^6^ CD4^+^ T cells)	
	D0	D42	D0	D42	D0	D42	D0	D42
10 μg H5VLP + alum	39.8	161.0**	23.8	128.5**	309.3	429.2*	131.7	142.5
15 μg H5VLP + alum	14.6	107.5**	27.4	101.8	362.5	426.6	77.2	158.4
20 μg H5VLP + alum	19.8	274.5**	40.3	99.3*	486.2	640.5	81.7	211.1
3.75 μg H5VLP + GLA	21.8	74.6**	13.8	80.9**	364.0	466.0	61.6	198.8**
7.5 μg H5VLP + GLA	10.9	51.5	20.4	44.9**	260.0	366.6	108.1	126.0
Placebo	21.5	9.2	21.1	16.1	389.2	260.5	41.7	62.0

Significant differences from D0 are indicated by *P ≤ 0.05, **P ≤ 0.01 or ***P ≤ 0.001 (Wilcoxon matched pairs signed-rank test)

#### Pre-boost Thpp frequency significantly correlated with serologic response 21 days after the boost (D42)

**Fig. 3:**
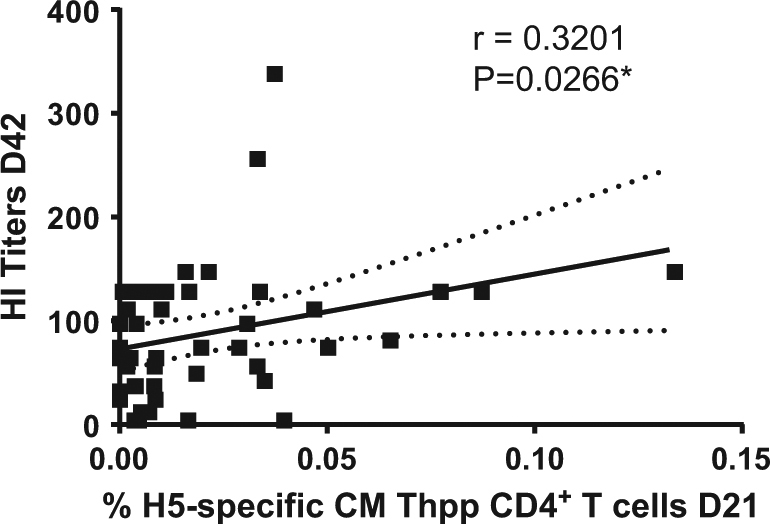
Correlation between the percent of H5-specific central memory (CM) CD45RA^−^ CD27^+^ Thpp (IL-2^+^ TNF-α^+^ IFN-γ^−^) CD4^+^ T cells at D21 (before boost) and the HI titer at D42 (21 days after boost) in vaccinated subjects. Spearman rank correlation coefficient *r* and *P* value are indicated (GraphPad, La Jolla, CA)

Although relatively low, the frequency of H5-specific CM Thpp CD4^+^ T cells at D21 significantly (*P* ≤ 0.01) correlated with HI titers at D42 in vaccinated groups regardless of the vaccine regimen (Fig. 3).

#### The adjuvanted H5VLP induced a significant polyfunctional and sustained CD4^+^ T cell heterologous response

**Fig. 4:**
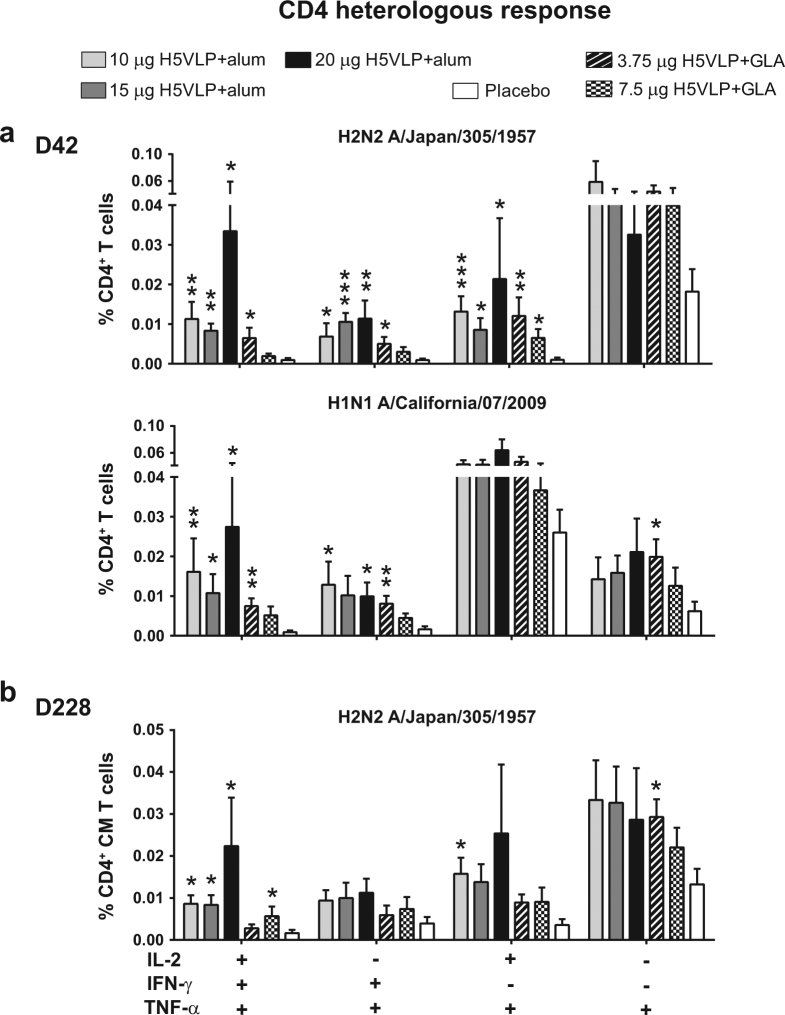
CD4^+^ T cell heterologous response. PBMCs were stimulated ex vivo with H2 peptide pool (**a** lower panel) or H1 peptide pool (**a** upper panel). The percentages (mean ± s.e.m) of H2-specific and H1-specific of the significantly impacted IL-2/IFN-γ/TNF-α CD4^+^ T cell functional signatures (detailed at the bottom of the figure) 42 days after prime are represented (**a**). The memory CD4^+^ T cell heterologous response was also measured 228 days after the prime (**b**). The percentages (mean ± s.e.m) of the significantly impacted IL-2/IFN-γ/TNF-α CD4^+^ central memory T cell functional signatures (detailed at the bottom of the figure) following ex vivo stimulation with H2 peptide pool are represented for the five vaccine regimens and Placebo. Asterisks indicate significant differences (*P ≤ 0.05), **P ≤ 0.01 or ***P < 0.001) from Placebo for each individual functional signature (Kruskal–Wallis test, followed by Dunn’s multiple comparisons test, GraphPad, La Jolla, CA)

Influenza hemagglutinin protein H2-specific TP CD4^+^ T cells significantly increased between D0 and D42 in H5VLP-vaccinated groups but not in Placebo (Table 2). The Thpp and TNF-α^+^/IFN-γ^+^(IL-2^−^) CD4^+^ T cells also significantly increased in all vaccinated groups with the exception of the 7.5 μg H5VLP + GLA (Table 2). All doses of the H5VLP vaccine induced higher proportions of cross-reactive H2-specific TP, IFN-γ^+^/TNF-α^+^(IL-2^−^) and Thpp CD4^+^ T cells as compared to Placebo 21 days after the boost with the exception of TP and IFN-γ^+^/TNF-α^+^(IL-2^−^) in the 7.5 μg H5VLP + GLA group (Fig. 4a, upper panel). Interestingly, this heterologous response occurred at the same order of magnitude as the H5-specific response. A similar cross-reactive response was observed for H1 although statistical significance was not reached for the Thpp CD4^+^ T cells (Fig. 4a, lower panel). Adjuvanted H5VLP vaccine only induced limited H7-specific cross-reactivity at D42, eliciting significantly higher SP TNF-α^+^ CD4^+^ T cells in the 7.5 μg H5VLP + GLA group as compared to Placebo (data not shown). Six months after the vaccination, H5VLP-vaccinated groups still exhibited higher proportion of polyfunctional H2-specific CD4^+^ CM T cells as compared to placebo (Fig. 4b). Notably, H2-specific TP CD4^+^ CM T cells were significantly higher in all H5VLP + alum and the 7.5 μg H5VLP + GLA groups. H2-specific Thpp and SP TNF-α^+^ CD4^+^ CM T cells were also higher than Placebo in vaccinated groups and this difference was statistically significant for the subjects who received two doses of 10 μg H5VLP + alum or 3.75 μg H5VLP + GLA, respectively (Fig. 4b).

## Discussion

All formulations of the H5VLP were well tolerated and no serious adverse event was observed confirming our previous observations.^6,14^ Antibody response, and especially HI titers, has long been and remains the major criteria driving the development and licensure of egg-based inactivated influenza vaccines. However, H5N1-inactivated vaccine has been demonstrated to be a poor HI titer inducer, requiring adjuvant and prime-boost strategy.^3,15^ All the H5VLP formulations tested in this study elicited an immune response. The alum-adjuvanted groups induced HI higher antibody titers than previously reported for a plant-produced recombinant H5 not self-assembling into a VLP^16^, and were similar or slightly higher than what we previously observed for the VLP vaccine^6,14^ and what was observed for an alum-adjuvanted-inactivated split virion vaccine.^17^ Antibodies induced by the plant-made H5VLP have previously been demonstrated to be neutralizing with low-to-moderate cross-reactivity.^6,14^ However, alum-adjuvanted groups only partially met the CHMP criteria confirming the limited impact of this adjuvant on the humoral response elicited by H5 vaccines, including H5VLP, in humans.^14,18,19^ By contrast, GLA-adjuvanted groups met all the CHMP criteria even at the lowest dose of 3.75 μg H5VLP in agreement with the significant impact of oil-in-water adjuvants, including MF59 and ASO3, observed with inactivated (split) virion vaccines,^20–22^ or with recombinant H5 protein.^23^ Although antigen deposition maintaining local antigen concentration was originally seen as the mechanism of action for aluminum salt adjuvants, more recent investigations demonstrated the role of inflammasome activation, release of endogenous danger signals, such as uric acid. On the other hand, the GLA-SE adjuvant, a TLR4 agonist formulated in a stable nano-emulsion of squalene oil-in-water, has been demonstrated to significantly increase the humoral response in animal models and human, as well as to drive Th1 response in vitro and in vivo.^24–26^ Interestingly Ko and co-workers^27^ recently demonstrated that alum adjuvants alone were unable to induce IgG antibodies against split vaccine in CD4KO mice after prime while monophosphoryl lipid A, another TLR4 agonist adjuvant, induced antigen-specific IgG1 in wildtype and CD4KO mice. However particular mechanisms of action specifically differentiating the different types of adjuvants remain largely unknown. While pre-formed Abs can be sufficient to provide protection from influenza, CMI is generally required to effectively clear viral infection and to maintain long-term immunity. Actually, there is a growing consensus that CMI plays a key role in long-term, cross-protective immunity to influenza.^10,13,28^ With the exception of the live-attenuated formulation (LAIV), commercial influenza vaccines are generally very poor inducers of robust anti-viral CMI.^29–31^ Very interestingly, both alum and GLA-adjuvanted H5VLP elicited comparable T cell response although the different doses of antigen would make difficult to compare between the two adjuvants.

We observed a massive (more than one order of magnitude) and sustained increase of H5-specific IL-2^+^/TNF-α^+^(IFN-γ^−^) CD4^+^ T cells 42 and 228 days after vaccination with both alum and GLA-adjuvanted H5VLP vaccine. This confirmed our previous observation of a substantial increase and predominance of this cytokine signature in H5-specific CD4^+^ T cells 6 months after immunization with an alum-adjuvanted H5VLP vaccine^14^ while revealing that this response actually takes place as soon as 21 days post boost. Influenza-induced IL-2^+^/TNF-α^+^(IFN-γ^−^) CD4^+^ T cells have been described as uncommitted Th1 Thpp preferentially promoted by recent, pandemic epitopes whereas common, multiply-boosted influenza epitope-specific CD4^+^ T cells expressed IFN-γ after influenza infections.^32,33^ Thpp have been proposed to serve as a reservoir of memory CD4^+^ T cells with effector potential.^33^ Our study further revealed that pre-boost Thpp response correlated with post-boost serological response enlightening the potential link between this particular T cells population and the B cell response. The frequency of HA-specific Thpp could therefore appear as an early marker for the subsequent antibody response. The plant-based H5VLP vaccine also induced a significant increase of homologous and cross-reactive TP polyfunctional CD4^+^ T cells. Polyfunctional T cells have been associated with better protection in several models,^34,35^ therefore representing an attractive immune cell population to promote after vaccination. The amplitude of cross-reactive CD4^+^ T cells observed against H1, H2 and to a lesser extent H7 was consistent with the sequence homologies of H5 with those HA proteins (74.5, 62.5 and 41.7% for H2, H1 and H7, respectively).^36^ As aforementioned, influenza history (infection or vaccination) and its relatively short exposure history may also explain why cross-reactive Thpp T cells responses tend to be higher for H1 than for the other HA strains. Although heterotypic immunity resulting from influenza infection appeared to be mainly provided by CD8 conserved epitopes of internal influenza proteins like nucleoprotein and M protein, recent data demonstrate that influenza HA molecule also contains class I-restricted and class II-restricted epitopes, and that HA-specific CD4^+^ T cell response can play significant role in cross-protection.^37,38^

No detectable CD8^+^ response was observed at D42. Although induction of major histocompatibility complex-I-restricted CD8^+^ response is best accomplished after endogenous expression of foreign proteins,^39^ dendritic cell-mediated cross-presentation and activation may occurred and has been observed in vitro*.*^40,41^ However the timing of sample collection as well as the length of peptides used for the ex vivo stimulation may have favored the detection of the CD4^+^ response. Ongoing studies are currently investigating the ability of plant-made VLP to stimulated CD8^+^ T cells.

In this study, we further characterized the previously observed long-term CMI elicited by the H5VLP.^14^ We observed that this long-term CD4^+^ T cell response was characterized by the presence of both CD27^−^ and CD27^+^ Ag-specific memory (CD45RA^−^) T cells. CD27 is a member of the TNF receptor family essential for the survival and accumulation of virus-specific T cells at the site of infection. Mice lacking CD27 are deficient in responding to T cell receptor stimulation and CD27^−/−^ transgenic mice displayed a reduced CD4^+^ and CD8^+^ T cell lung infiltration during influenza infection as compared to wild-type.^42,43^ Lung homing T cells and their proliferation ability have been demonstrated as important correlates of vaccine protection against influenza in mice^44^ and circulating CD4^+^ memory cells directed against influenza in human show high expression of CD27^45^ with a high proliferative potential and a good ability to provide help to B cells.^46^ The memory response was also altered in CD27^−/−^ transgenic mice demonstrating the role of CD27 for generation and long-term maintenance of CMI. Interestingly, the long-term heterologous H2-specific CD4^+^ T cells response were mainly CD27^+^ memory cells. Contrasting with the major impact on the humoral immune response, the CMI was marginally influenced by the nature of the adjuvant indicating intrinsic effects of the VLP on T cell response.

In addition to the strong humoral response elicited by the GLA-adjuvanted H5VLP vaccine, we also observed a sustained and cross-reactive CD4^+^ T cell response regardless of the adjuvant nature. While egg-based inactivated influenza vaccine are prone to elicit strong humoral response (with the aforementioned restriction for H5), those vaccine are generally poor inducers of robust CMI as opposed to live-attenuated influenza vaccine (LAIV), which induced substantial T cells response^29–31^ but may run the risk of recombination with circulating strains. Immune protection against influenza infection rely on complex interplay, where CD4^+^ T cells have been demonstrated to provide significant help to influenza virus-specific Abs, B cell response and CD8^+^ T cytotoxic response.^11,28,47^ Additionally, Wilkinson and co-workers reported that preexisting influenza-specific CD4^+^ T cells correlate with disease protection against influenza challenge in humans.^48^ The protection provided by the plant-based VLP vaccine in animal models, including cross-clade protection^4,6,8^ likely lies at least partially in its ability to adequately stimulate both arms of the immune response.

## Methods

### Study design and participants

The phase II trial was a randomized (1:1:1:1:1:1) multicenter, double-blind, placebo-controlled, dose-ranging study (NCT01991561)^49^ conducted in Canada at the McGill University Health Centre (Montreal, QC) and the INC Research Centre (Toronto, ON) between June 2013 and September 2014. The study was carried out in accordance with the Declaration of Helsinki and the principles of Good Clinical Practices and was approved by the site’s Ethics Review Board and by the Center for Biologics Evaluation and Research (CBER). Written consent was obtained from all study participants. The randomization was stratified by site and was generated by Veristat (Holliston, MA) using random block permutations obtained with the PLAN procedure from SAS^®^ software, version 9.2 (Cary, NC). Allocation was evenly split across the two sites and each site provided a pre-determined sequence of randomization numbers according to the randomization code. The objectives were to evaluate safety, tolerability and immunogenicity of two doses of adjuvanted H5VLP administered intramuscularly (IM). The study included 390 healthy adults of 18–60 years of age with BMI ≥18 and ≤32. The other inclusion and exclusion criteria are detailed at Clinicaltrials.gov^49^ and in the detailed protocol (Suppl. Material).

### Procedures

Two hundred-five women and 185 men were allocated into one of the six groups who received two doses (prime-boost) by IM 21 days apart of 10, 15 or 20 µg of H5VLP combined with either 500 µg Alhydrogel^TM^ adjuvant (H5VLP + alum), 3.75 or 7.5 µg of H5VLP combined with GLA-SE (H5VLP + GLA) or phosphate-buffered saline placebo (65 subjects/group). GLA-SE is a stable oil-in-water emulsion consisting of squalene, glycerol, phosphatidylcholine, polaxamer surfactant and ammonium phosphate buffer at a final concentration of 5 μg GLA/2% SE (w/v) per vaccine dose. Solicited local and general symptoms were recorded 7 days following each vaccine dose. Serum and blood samples were obtained at D0, D21, D42 (21 days after the boost) and D228 after prime for HI assay and T cell response against homologous and heterologous strains.

### The vaccine

Production of the H5VLP was based on HA sequence of A/Indonesia/05/2005 H5N1 influenza strain as previously described.^5,8^

### HI assay

HI assay was performed as previously described according to the WHO recommendation.^6,50^ Due to their high pathogenicity, handling live wild-type H5N1 strains require Biosecurity Level 3 facility. The H5VLP was therefore used as surrogate reagent to perform HI assay. The SCR, SPR and GMFR were defined according to regulatory criteria (CPMP, 1997)^51^ and were compared to the CHMP criteria i.e., SCR ≥ 40%, SPR ≥ 70%, GMFR ≥ 2.5 for healthy adults.

### CMI

The T cell response was assessed in ten subjects/group at D0, D21, D42 and D228 as previously described.^14^ Briefly, peripheral blood mononucleated cells (PBMC) were stimulated ex vivo with 2.5 μg/ml of peptide pool consisting of 15mer peptides overlapping by 11 amino acids spanning the complete HA of the homologous A/Indonesia/05/2005 H5N1 (H5) or heterosubtypic A/Japan/305/1957 H2N2 (H2), A/California/07/2009 H1N1 (H1) or A/Hangzhou/1/2013 H7N9 (H7) strains (GenScript, Piscataway, NJ). The markers and the antibodies used for the flow cytometry analysis are detailed in Suppl. Table 2. The data acquisition was performed on BD LSRII flow cytometer (Becton Dickinson, Franklin Lakes, NJ). Approximately 3 × 10^5^ viable lymphocytes were acquired for each sample and data were analyzed using FlowJo™ v9.7 (Tree Star, OR), Pestle v1.7 and SPICE v5.2 (Mario Roederer, Vaccine Research Centre, National Institutes of Health, USA, available at http://exon.niaid.nih.gov/spice) softwares. The gating strategy is detailed in Suppl. Figure 2. The total cell viability was always >85%. SPICE-based functional analysis was performed on background-subtracted values from non-stimulated PBMC.

### Statistical analysis

The sample size of 65 subjects per group was based on previously published results^6,14^ and on the probability of meeting CHMP criteria. Analysis of variance (ANOVA) and Fisher’s exact tests were respectively used for treatment comparisons of continuous variables and proportions of the demographics and baseline data (SAS^®^ software, version 9.2). Fisher’s exact test was also used to compare the occurrence of solicited symptoms between vaccinated subjects and Placebo (Prism™ Software v6.0, GraphPad, La Jolla, CA). Differences in CD4^+^ T cell response over the time in the vaccinated groups and Placebo were analyzed by two-way ANOVA followed by a Dunnett’s multiple comparison post hoc analysis. The increase of frequencies between D0 and D42 for each CD4^+^ subpopulations characterized by their cytokine signature was addressed by a Wilcoxon matched-pairs signed rank test (Prism™ Software v6.0, GraphPad, La Jolla, CA). A Kruskal–Wallis test, followed by Dunn’s multiple comparison post hoc analysis were conducted independently for each adjuvant type to identify the significant differences between vaccinated groups and Placebo at different time-points (Prism™ Software v6.0, GraphPad, La Jolla, CA). Correlations between CMI and humoral responses were addressed by the Spearman rank correlation test (Prism™ Software v6.0, GraphPad, La Jolla, CA). Differences with a *P* value < 0.05 were considered significant.

### Data availability

Clinical data that support the findings of this study are available from Medicago Inc. but restrictions apply to the availability of these data. Data are, however, available from the authors upon reasonable request and with permission of Medicago Inc. To protect the privacy of patients and individuals involved in our studies, Medicago Inc. does not publically disclose patient-level data.

## Electronic supplementary material

Supplementary Table 1

Supplementary Table 2

Supplementary Figure 1

Supplementary Figure 2
