# Supplementary material for: Humoral and cell-mediated immune responses to H5N1 plant-made virus-like particle vaccine are differentially impacted by alum and GLA-SE adjuvants in a Phase 2 clinical trial
Source: NPJ Vaccines. 2018 Jan 23;3:3. doi: 10.1038/s41541-017-0043-3 (PMC5780465; doi:10.1038/s41541-017-0043-3)
Supplement: Supplementary file 1 — Supplementary Table 1 [file 41541_2017_43_MOESM1_ESM.docx]

**Suppl. Table 1: Demographics and baseline characteristics**

| **Parameter** | **H5 VLP+alum** | | |  | **H5 VLP+GLA-SE** | | **Placebo** | **Total** | **P-value^1^** |
| --- | --- | --- | --- | --- | --- | --- | --- | --- | --- |
|  | **10 µg** | **15 µg** | **20 µg** |  | **3.75 µg** | **7.5 µg** |  |  |  |
| **Age (years)** |  |  |  |  |  |  |  |  |  |
| N | 65 | 65 | 65 |  | 65 | 65 | 65 | 390 | 0.8409 |
| Mean (Standard deviation) | 37.0 (13.65) | 36.6 (13.71) | 37.8 (12.91) |  | 36.5 (14.05) | 38.6 (12.59) | 35.0 (13.85) | 36.9 (13.43) |  |
| Median | 34 | 36 | 38 |  | 33 | 40 | 31 | 37.5 |  |
| Min, Max | 18, 59 | 18, 59 | 18, 60 |  | 18, 59 | 18, 60 | 18, 59 | 18, 60 |  |
| **Gender, N (%)** |  |  |  |  |  |  |  |  |  |
| Female | 35 (53.8) | 32 (49.2) | 36 (55.4) |  | 31 (47.7) | 40 (61.5) | 31 (47.7) | 205 (52.6) | 0.6933 |
| Male | 30 (46.2) | 33 (50.8) | 29 (44.6) |  | 34 (52.3) | 25 (38.5) | 34 (52.3) | 185 (47.4) |  |
| **Race, N (%)** |  |  |  |  |  |  |  |  |  |
| Caucasian | 57 (87.7) | 48 (73.8) | 50 (76.9) |  | 52 (80.0) | 46 (70.8) | 54 (83.1) | 307 (78.7) | 0.2835 |
| Asian | 4 (6.2) | 7 (10.8) | 10 (15.4) |  | 8 (12.3) | 10 (15.4) | 7 (10.8) | 46 (11.8) |  |
| Black or African American | 4 (6.2) | 6 (9.2) | 4 (6.2) |  | 5 (7.7) | 8 (12.3) | 3 (4.6) | 30 (7.7) |  |
| Other | 0 | 4 (6.2) | 1 (1.5) |  | 0 | 0 | 1 (1.5) | 6 (1.5) |  |
| Native American/Alaskan | 0 | 0 | 0 |  | 0 | 1 (1.5) | 0 | 1 (0.3) |  |
| **Body mass index (kg/m^2^)** |  |  |  |  |  |  |  |  |  |
| Mean (Standard deviation) | 25.1 (3.22) | 24.9 (2.98) | 25.7 (3.91) |  | 25.8 (3.12) | 25.6 (3.40) | 24.6 (3.55) | 25.3 (3.39) | 0.3471 |
| Median | 25 | 25 | 26 |  | 26 | 25 | 24 | 25 |  |
| Min, Max | 18, 31 | 19, 32 | 18, 32 |  | 20, 32 | 19, 32 | 19, 32 | 18, 32 |  |
| **Influenza vaccination history, N (%)** |  |  |  |  |  |  |  |  |  |
| Seasonal, Avian, or Combo | 14 (21.5) | 17 (26.2) | 14 (21.5) |  | 18 (27.7) | 21 (32.3) | 14 (21.5) | 98 (25.1) | 0.7599 |
| Reaction to vaccination | 1 (7.1) | 4 (23.5) | 4 (28.6) |  | 3 (16.7) | 3 (14.3) | 4 (28.6) | 19 (19.4) | 0.7472 |
| No Vaccination | 51 (78.5) | 48 (73.8) | 51 (78.5) |  | 47 (72.3) | 44 (67.7) | 51 (78.5) | 292 (74.9) |  |

^1^Fisher’s exact test was used for treatment comparison of the proportion data; ANOVA was used for treatment comparison of the continuous variables. *P*-value for race compares Caucasian vs other races.
