# Supplementary material for: Humoral and cell-mediated immune responses to H5N1 plant-made virus-like particle vaccine are differentially impacted by alum and GLA-SE adjuvants in a Phase 2 clinical trial
Source: NPJ Vaccines. 2018 Jan 23;3:3. doi: 10.1038/s41541-017-0043-3 (PMC5780465; doi:10.1038/s41541-017-0043-3)
Supplement: Supplementary file 2 — Supplementary Table 2 [file 41541_2017_43_MOESM2_ESM.docx]

**Suppl. Table 2: Immunological markers used for the flow cytometry analysis**

| **Markers** | **Fluorochromes** | **(Clones) Manufacturers** |
| --- | --- | --- |
| Live/dead | V500 | Invitrogen |
| CD3 | V450 | (SP34-2) BD Horizon |
| CD4 | Phycoerythrin-TexasRed | (SFCI2T4D11) Beckman Coulter |
| CD8 | eFluor 650 NC | (RPA-T8) eBioscience |
| CD27 | Brilliant violet 605 | (O323) BioLegend |
| CD45RA | Allophycocyanin-Cyanine7 | (HI100) BioLegend |
| IL-2 | Alexa 700 | (MQ1-17H12) BioLegend |
| IFN-γ | Phycoerythrin-Cyanine7 | (B27) BD Pharmingen |
| TNF-α | Allophycocyanin | (6401.1111) BD Biosciences |
| Granzyme B | Fluorescein isothiocyanate | (GB11) BD Pharmingen |
| Perforin | Phycoerythrin | (B-D48) BioLegend |
| CD107a | PerCP-Cyanine5.5 | (H4A3) BioLegend |
